# Drug repurposing applied: Activity of the anti-malarial mefloquine against *Echinococcus multilocularis*

**DOI:** 10.1016/j.ijpddr.2020.06.002

**Published:** 2020-07-02

**Authors:** Britta Lundström-Stadelmann, Reto Rufener, Andrew Hemphill

**Affiliations:** Institute of Parasitology, Department of Infectious Diseases and Pathobiology, Vetsuisse Faculty, University of Bern, Längggassstrasse 122, 3012, Bern, Switzerland

**Keywords:** Alveolar echinococcosis, Metacestode, MMV665807, Niclosamide, Atovaquone, Energy-metabolism

## Abstract

The current chemotherapeutical treatment against alveolar echinococcosis relies exclusively on benzimidazoles, which are not parasiticidal and can induce severe toxicity. There are no alternative treatment options. To identify novel drugs with activity against *Echinococcus multilocularis* metacestodes, researchers have studied potentially interesting drug targets (e.g. the parasite's energy metabolism), and/or adopted drug repurposing approaches by undertaking whole organism screenings. We here focus on drug screening approaches, which utilize an *in vitro* screening cascade that includes assessment of the drug-induced physical damage of metacestodes, the impact on metacestode viability and the viability of isolated parasite stem cells, structure-activity relationship (SAR) analysis of compound derivatives, and the mode of action. Finally, once *in vitro* data are indicative for a therapeutic window, the efficacy of selected compounds is assessed in experimentally infected mice. Using this screening cascade, we found that the anti-malarial mefloquine was active against *E. multilocularis* metacestodes *in vitro* and *in vivo*. To shed more light into the mode of action of mefloquine, SAR analysis on mefloquine analogues was performed. *E. multilocularis* ferritin was identified as a mefloquine-binding protein, but its precise role as a drug target remains to be elucidated. In mice that were infected either intraperitoneally with metacestodes or orally with eggs, oral treatment with mefloquine led to a significant reduction of parasite growth compared to the standard treatment with albendazole. However, mefloquine was not acting parasiticidally. Assessment of mefloquine plasma concentrations in treated mice showed that levels were reached which are close to serum concentrations that are achieved in humans during long-term malaria prophylaxis. Mefloquine might be applied in human AE patients as a salvage treatment. Future studies should focus on other repurposed anti-infective compounds (MMV665807, niclosamide, atovaquone), which showed stronger *in vitro* activity against *E. multilocularis* than mefloquine.

## Highlights

• Anti-infective drugs are repurposed for identification of novel drugs against AE.
• Mefloquine is active against *E. multilocularis in vitro* and in mice.
• The energy metabolism of *E. multilocularis* is a target for future drug development.
• MMV665807 and niclosamide are highly active against *E. multilocularis in vitro.*

## Graphical abstract

**Figure:**
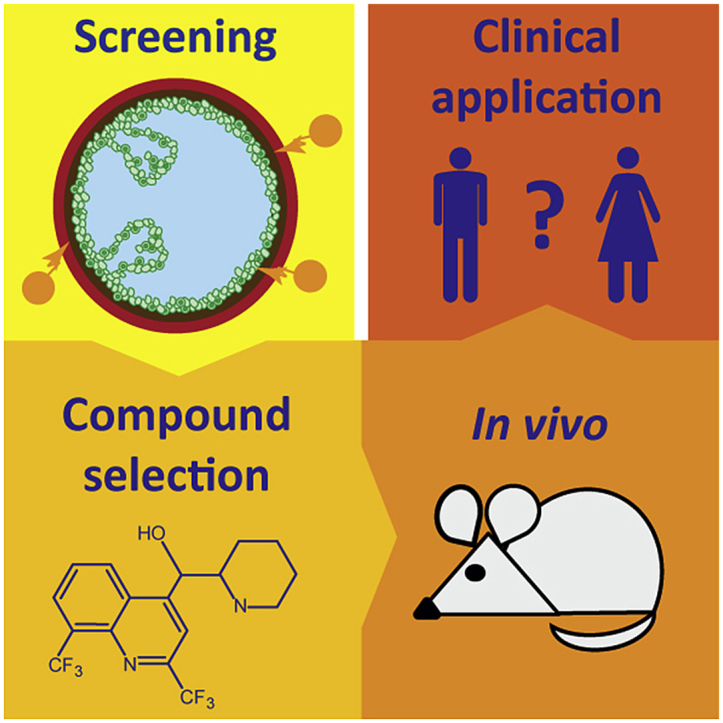

## Abbreviations

ABZ: albendazole
AE: alveolar echinococcosis
BMZ: benzimidazole
DALYs: disability adjusted life years
ELQ: endochin-like quinolone
GL: germinal layer
LL: laminated layer
MBZ: mebendazole
MD: malate dismutation
MMV: Medicines for Malaria Venture
SAR: structure-activity relationship
TDH: threonine dehydrogenase

## 1 Introduction – alveolar echinococcosis (AE)

### 1.1 AE - why should we care?

Alveolar echinococcosis (AE) is caused by the cestode *Echinococcus multilocularis* (small fox tapeworm). AE is the highest ranked foodborne parasitic disease in Europe and is classified third on a global level (Bouwknegt et al., 2018), despite relatively low case numbers in comparison to other parasitic diseases. In 2010, 18′451 new human cases of AE were estimated (Torgerson et al., 2015), with the highest prevalences in Asia with up to 9.43% in Qinghai, China, and 6.4% in Kyrgyz Alay district, Kyrgyzstan (Baumann et al., 2019; Bebezov et al., 2018). In Western-Central Europe, the annual infection rate is comparably low with 0.3–3 per 1′000′000 inhabitants (Gottstein et al., 2015a). However, due to the severity and fatality of the disease, human AE has an annual global impact of more than 687′823 disability adjusted life years (DALYs) (Torgerson et al., 2015), which poses an uncontrolled health problem especially in developing and resource-poor regions (Kern et al., 2017). Furthermore, AE is recognized as an emerging disease in Europe, North America and Asia (Gottstein et al., 2015a; Thompson, 2017; Trotz-Williams et al., 2017; Bebezov et al., 2018; Robertson, 2018; Polish et al., 2020), and curative drug treatments are still lacking (Lundström-Stadelmann et al., 2019).

### 1.2 *E. multilocularis* and AE – how we get infected

*E. multilocularis* is endemic in the Northern hemisphere and found all over Central and Eastern Europe, Central and Eastern Asia, and North America. The natural life cycle of *E. multilocularis* includes definitive hosts such as canids (foxes, dogs, and raccoon-dogs) and a variety of mammalian intermediate hosts (mostly rodents, but accidently also humans, captive monkeys, dogs, and others) (Romig et al., 2017). Intermediate or accidental hosts get orally infected through parasite eggs that are shed within the faeces of final hosts and contaminate the environment. Each egg contains a first larval stage, the oncosphere, which is released in the intestinal lumen of the intermediate host. The oncosphere then migrates through the intestinal wall, reaches the bloodstream and ends up primarily in the liver, where it develops into the second larval stage, the metacestode, and thereby causes the disease AE. In susceptible hosts, the metacestode undergoes unlimited asexual proliferation by budding of parasite vesicles, and thus grows infiltratively into the surrounding host tissue. *E. multilocularis* metacestodes form cancer-like lumps, and can also form metastases at more distant sites of the body. Eventually, AE results in severe organ dysfunction, mostly in the liver, but also in other infected organs. In natural hosts, but rarely in humans, brood capsules with protoscoleces are formed within the metacestodes after several months. These represent precursors of the next generation of tapeworms. To conclude the parasite life cycle, ingestion of a naturally infected rodent by a final host results in attachment of protoscoleces to the intestinal wall of the final host, followed by the development of adult tapeworms (Romig et al., 2017).

In human patients, AE is a chronic disease, which exhibits severe symptoms that appear usually 10–15 years post-infection. It has many pathological similarities with a slowly growing, malignant hepatic tumor. The parasitic lesion gradually invades the liver tissue, vessels, and bile ducts and may reach a size of up to 20 cm with a central necrotic cavity (Kern et al., 2017). In the progressive stage of human AE, non-specific symptoms such as abdominal pain, jaundice, cholestasis, hepatomegaly, fever, anaemia, weight loss, and pleural pain appear (Kern et al., 2017). Additionally, an AE diagnosis poses a continuously high psychological burden to patients (Schmidberger et al., 2018; Nikendei et al., 2019). Finally, at an advanced stage, and if not treated properly or if treatment fails, AE will lead to the death (Lundström-Stadelmann et al., 2019).

It has been hypothesized that, at least in Switzerland, only a small fraction of people exposed to infectious *E. multilocularis* eggs develop a progressive form of the disease (Gottstein et al., 2015b). The reasons for this are still unknown, but the immune system is crucial in determining the final outcome of infection. Several studies reported on the rapid development of AE in HIV patients (Sailer et al., 1997; Zingg et al., 2004), and others have brought up the increased risk of occurrence and progression of AE in immunosuppressed patients (Chauchet et al., 2014; Gottstein et al., 2015b; Lachenmayer et al., 2019).

## 2 AE – current drugs and treatments

Invasive surgical resection of the whole parasite tissue represents the only curative treatment of AE. However, this cannot be achieved at later stages of infection, when the parasite has grown and spread highly invasively (Grüner et al., 2017; Salm et al., 2019). Therefore, radical surgery is only applied in 20%–50% of all human AE cases (Kern et al., 2017). Surgery always has to be combined with temporal chemotherapy and long-term monitoring to treat and follow-up eventual parasite residues (Kern et al., 2017). If such a complete surgical resection of the parasite is not feasible, treatment relies exclusively on the benzimidazoles (BMZs) mebendazole (MBZ, 40–50 mg/kg body weight, three daily doses) or albendazole (ABZ, 10–15 mg/kg body weight, two daily doses) (Brunetti et al., 2010). The parasitostatic action of BMZs reduces, in the best case, further growth of the parasite. The described mode of action of BMZs involves the binding to beta-tubulin (resulting in the inhibition of tubulin polymerization and all connected cellular processes), a target which is expressed in the stem cells of *E. multilocularis* metacestodes as an isoform with low affinity to these drugs (Brehm and Koziol, 2014). Therefore, stem cells of *E. multilocularis* are more resistant to BMZs than other cells of the parasite, and this leads, together with the limited uptake and half-life of BMZs, to a parasitostatic rather than parasiticidal effect. The consequence of this is that for the treatment of AE, BMZs have to be taken life-long as parasite growth will resume its growth upon treatment discontinuation. The multiple daily dosages of BMZs in AE patients lead to problems in compliance, as well as to side-effects (in 54.5% of patients from a study in Germany), including severe adverse effects (described in 6.9% of German AE patients) leading to treatment discontinuation and no other options left for further treatment (Grüner et al., 2017). Disease progression due to treatment failure was described in up to 16% of German AE cases (Grüner et al., 2017). In countries with lower access to good health infrastructure, close monitoring of drug-levels might not be feasible, and thus adverse effects and treatment failures might be more common but remain unreported. All these shortcomings underline the urgency in developing alternative chemotherapeutic options against AE.

## 3 Identification of novel drugs against alveolar echinococcosis

### 3.1 *In vitro* and *in vivo* drug testing models

**Fig. 1:**
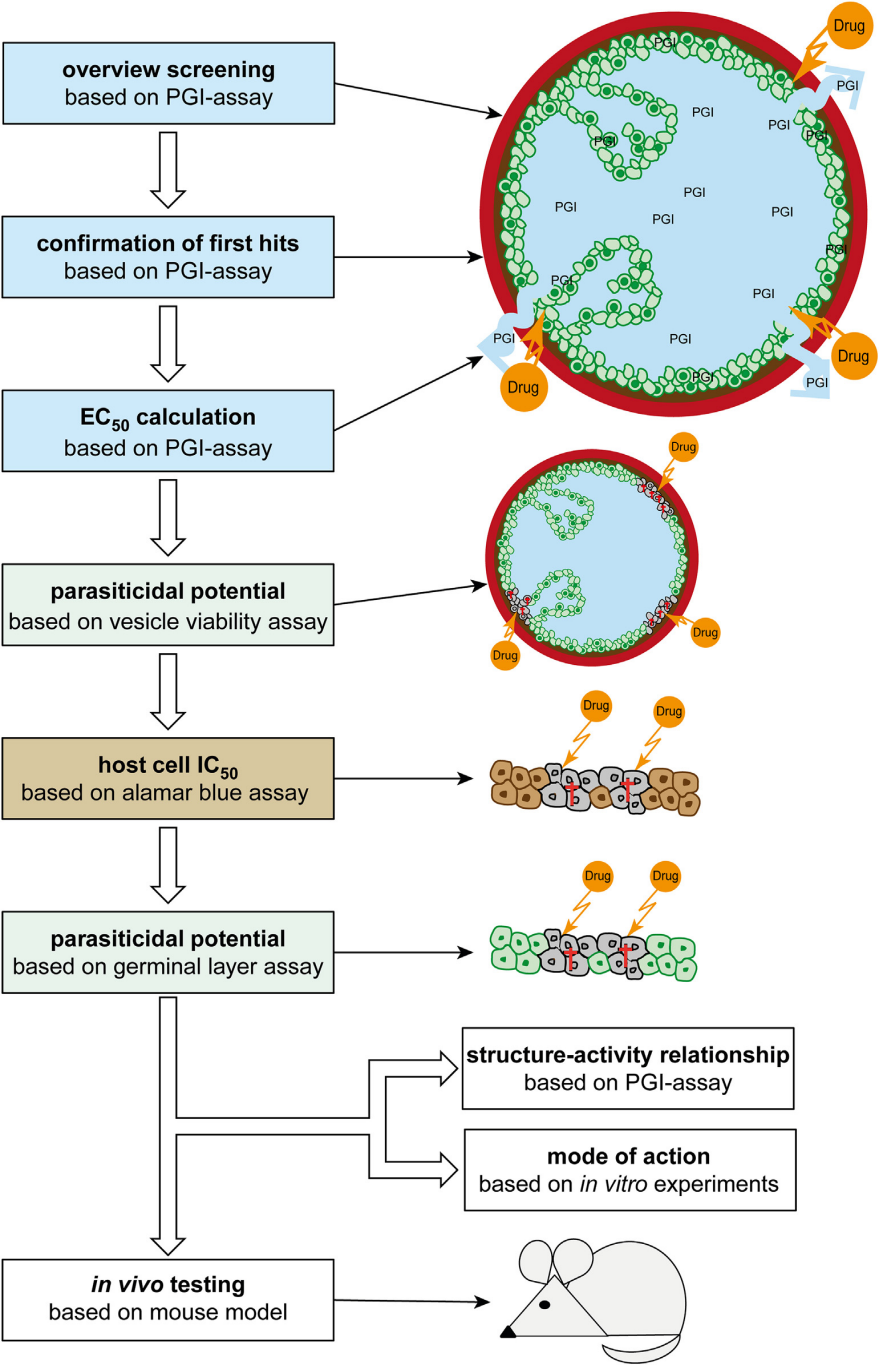
***In vitro* drug-screening pipeline to identify novel compounds against *E. multilocularis***. The pipeline can be applied to screen drug collections containing up to several hundred molecules to identify compounds with distinct activity against *E. multilocularis* metacestodes. First, overview screenings are performed on whole metacestodes by measuring damage marker release in the PGI-assay. Positive hits are confirmed, and EC_50_ values calculated based on the same assay. The parasiticidal potential is assessed on metacestodes by a viability assay (Alamar blue assay). Thereafter, toxicity on various mammalian cell lines is assessed (Alamar blue assay). The parasiticidal potential is further confirmed by assessing the viability of isolated GL cells (CellTiter Glo assay). If all the above-mentioned tests are indicative for a therapeutic window, more in-depth studies concerning the mode of action and SAR studies can optionally be pursued. Finally, compounds are assessed in experimentally infected mice. Figure adapted from (Lundström-Stadelmann et al., 2019).

Several years ago, markedly improved *in vitro* culture techniques for *E. multilocularis* metacestodes and primary (stem) cells were made available (Spiliotis et al., 2004, 2008). The *in vitro* grown metacestodes exhibit the same morphological features as naturally grown metacestodes: each metacestode consists of a cluster of fluid-filled vesicles, which are surrounded by three layers: the laminated layer (LL), the tegument, and the germinal layer (GL). The acellular carbohydrate-rich LL, which forms the outer surface, protects the parasite against host reactions. The syncytial tegument is anchored to the interior surface of the LL via microvilli-like protrusions termed microtriches, and is followed by the GL, which is composed of connective tissue, muscle cells, nerve cells, glycogen storage cells, and undifferentiated stem cells (Koziol et al., 2014). These stem cells need to be killed to achieve a parasiticidal effect upon drug treatment of the parasite (Brehm and Koziol, 2014). Based on these *in vitro* grown *E. multilocularis* metacestodes, different drug-screening assays were developed, which produced objective and quantifiable read-outs that were all combined in a screening pipeline to identify active compounds from a collection of diverse molecules (see Fig. 1) (Lundström-Stadelmann et al., 2019). These include (i) PGI-assay (Stadelmann et al., 2010), with PGI release as an indicator of physical impairment of metacestodes; (ii) Alamar blue assay (Stadelmann et al., 2016), which measures loss of viability of metacestodes; (iii) CellTiter Glo assay to assess ATP production and thus viability of isolated parasite stem cells (Stadelmann et al., 2016); (iv) electron microscopy to determine morphological/structural effects (Stadelmann et al., 2016; Rufener et al., 2018a); in addition (v) Alamar blue assay is performed on drug treated mammalian cell cultures to determine a potential therapeutic window (Rufener et al., 2018a); and (vi) structure-activity relationship (SAR) studies are carried out with several derivatives from one compound (Rufener et al., 2018b), which contribute to (vii) investigations on the mode of action (Rufener et al., 2018a). Finally, if *in vitro* data are indicative for a therapeutic window, (viii) the efficacy of selected compounds is assessed in experimentally infected mice. Although not representing the exact natural intermediate hosts, laboratory mice are very close to natural intermediate hosts of *E. multilocularis*, and they allow to study the parasite in a controlled *in vivo* setting. There are two murine models that are commonly applied: (i) the secondary infection model, in which mice are intraperitoneally infected with *E. multilocularis* metacestode suspension. This model represents the advanced, disseminated and chronic stage of AE (Stadelmann et al., 2016; Gorgas et al., 2017); (ii) the primary egg infection model (Rufener et al., 2018b), in which mice are orally infected by gavage of *E. multilocularis* eggs, representing the natural route of infection and early parasite lesions.

### 3.2 Drug development against AE

As in other fields of drug development, two basic approaches can be followed to identify novel compounds with efficacy against AE: (i) mechanism-/target-based screening (bottom-up approach), or (ii) phenotypic screening of whole organisms (top-down approach) (Geary et al., 2015; Müller and Hemphill, 2016; Aulner et al., 2019). For target-based screening, a valid molecular target in the parasite has to be identified and validated, before specific inhibitors are designed and screened. Even though this approach has not yet been successful in bringing anti-parasitic drugs on the market, it is generally seen as the more sophisticated approach. An essential prerequisite for the identification of novel targets is a profound knowledge of the parasite biochemistry and its metabolic requirements. A recent publication by Ritler et al. (2019) reported on the molecular footprint of *E. multilocularis* metacestodes, and by analysing the metabolic host-parasite interface *in vitro*, a number of potentially targetable metabolic pathways were identified. An important finding was that not only the ubiquitous energy source glucose, but also the amino acid threonine, was consumed by the parasite at high levels. Threonine consumption could involve the catabolism of the amino acid through the threonine dehydrogenase (TDH), which is described in several other organisms, but is inactive in humans (Edgar, 2002; Wang et al., 2009; Millerioux et al., 2013), thus representing a potential target to be followed in the future. In addition, Ritler et al. showed that final electron acceptors such as succinate, acetate, and fumarate were released in by the metacestodes at high amounts, which is strongly indicative that the malate dismutation (MD) pathway is functionally active in these parasites (McManus and Smyth, 1978). MD is found in helminths, marine invertebrates, and euglenids, but is absent in mammals. It basically represents an inversion of the TCA cycle, using rhodoquinone instead of ubiquinone as an electron carrier (Tielens, 1994; Komuniecki and Harris, 1995; Kita et al., 2003). The MD pathway allows for mitochondrial energy generation under anaerobic conditions (Bryant, 1970; Tielens, 1994). As already primarily investigated in *Echinococcus* protoscoleces and other helminths, the MD of *E. multilocularis* could represent a viable drug target (Tielens, 1994; Matsumoto et al., 2008).

Recently, two studies reported on the successful inhibition of the electron transfer chain in *E. multilocularis* GL cells and metacestodes by atovaquone, buparvaquone, and the endochin-like quinolone ELQ-400 (Rufener et al., 2018a; Enkai et al., 2020). Thus, the respective target, the complex III of the mitochondrial respiration chain, could be further followed up with more selective inhibitors. In conclusion, the energy metabolism of *E. multilocularis* is different from the mammalian one, and this could offer different options for a targeted development of novel drugs.

Phenotypic screening of whole organisms has so far been the approach which led to all anti-parasitic drugs on the market (Aulner et al., 2019). For *E. multilocularis* and AE, the methods described in 3.1. can be applied for such screenings (Lundström-Stadelmann et al., 2019). Due to the fact that pharmaceutical companies have been increasingly reluctant to engage in preclinical drug development for AE, the focus is on repurposing of already existing drugs or compound classes that are on the market or being developed for other indications. Marketed drugs have the highest potential for reaching AE patients responding poorly to benzimidazoles, or with major side-effects, to apply them as salvage treatment. Moreover, drug-repurposing also includes experimental molecules that have proven activities against different indications, albeit their pharmacological profile is not completely described yet or not suitable for clinical application in patients. Nevertheless, such drugs are a promising starting point for screening approaches with limited resources, and potentially allow to gain more insights about their possible modes of action and thus potentially valuable targets where research should be focused. Most studies on drug repurposing against AE evaluated an overall relatively small number of compounds, including anti-cancer compounds (Naguleswaran et al., 2006; Gelmedin et al., 2008, 2010; Spicher et al., 2008a; Hübner et al., 2010; Hemer and Brehm, 2012; Schubert et al., 2014; Huang et al., 2018; Joekel et al., 2018; Förster et al., 2019), anti-infective drugs (Reuter et al., 2003, 2006; Stettler et al., 2003; Spicher et al., 2008b; Stadelmann et al., 2010, 2011; Küster et al., 2011, 2014a, 2014b, 2015; Rufener et al., 2018a, 2018b), immunotherapeutics (Emery et al., 1998; Pfister et al., 1989; Godot et al., 2003; Boubaker et al., 2015; Wang et al., 2018; Jebbawi et al), and natural products (Albani Clara and Elissondo María, 2014; Albani et al., 2015; Yuan et al., 2016; Hizem et al., 2019). In the following sections, we will focus more on anti-infective drugs, and in particular on the group of anti-malarials.

#### 3.2.1 Repurposing of anti-infective drugs against AE

A plethora of anti-infective compounds were tested against *E. multilocularis* in rodent models and *in vitro* in the past (Lundström-Stadelmann et al., 2019), including (i) BMZ (Siles-Lucas et al., 2018), (ii) nitazoxanide (Stettler et al., 2003), (iii) amphotericin B and itraconazole (Reuter et al., 2003, 2006), (iv) inhibitors of the mitochondrial respiration chain (Enkai et al., 2020; Rufener et al., 2018a), and (v) mefloquine (further discussed in chapter 4 and following).

However, to date only the (i) BMZ were shown to be active also against human AE. More recent studies suggest to improve the absorption and oral bioavailability of these drugs by developing new formulations such as BMZ salt formulations (Cirilli et al., 2017) or nanocrystals (Pensel et al., 2018; Bakhtiar et al., 2019; Ullio Gamboa et al., 2019). Two other drugs that reached clinical application against AE over the last years are (ii) the broad-spectrum anti-parasitic nitazoxanide and (iii) the anti-fungal agent amphotericin B. However, they were not further pursued due to low or no activity in humans, and also pronounced side-effects (Kern et al., 2008; Tappe et al., 2009). (iv) Inhibitors of the mitochondrial respiratory chain (i.e. buparvaquone, atovaquone and the endochin-like quinolone ELQ-400) were identified as highly active compounds *in vitro* (Rufener et al., 2018a; Enkai et al., 2020). Atovaquone exhibited activity in the primary mouse infection model against AE (Enkai et al., 2020), whereas buparvaquone was not active in the secondary AE mouse infection model, presumably due to the fast metabolization and short plasma half-life of buparvaquone (Rufener et al., 2018a). Further analyses revealed that buparvaquone, atovaquone, and most likely also ELQ-400, inhibit the mitochondrial complex III in *E. multilocularis* (Rufener et al., 2018a; Enkai et al., 2020). These inhibitors of the respiratory chain will have to be investigated further to determine whether they can be translated into a promising treatment option against human AE.

## 4 Mefloquine

Mefloquine is an anti-malarial drug, which is structurally related to quinine. Mefloquine is known to induce various side-effects, ranging from diarrhea, nausea, and stomach pain, to more severe adverse reactions such as neurological and psychological disturbances (Tickell-Painter et al., 2017). However, the high half-life of mefloquine allows for less frequent dosing in malaria-prophylaxis, and thereby better patient compliance. For these reasons, the use of mefloquine is mostly limited for the prevention and treatment of chloroquine-resistant malaria and for prophylaxis in pregnant malaria patients, but mefloquine is not recommended for patients with a previous history of psychological disorders (Tickell-Painter et al., 2017).

Mefloquine is not only active against *Plasmodium*, but also against a range of trematode species: it was shown to exhibit promising activity against schistosomiasis *in vitro* and in mice (Keiser et al., 2009a; Manneck et al., 2010; Panic et al., 2017), and against *S. haematobium* infection in humans (Keiser et al., 2010; Basra et al., 2013), but did not increase the efficacy of praziquantel when tested in humans with *S. haematobium* infections (Keiser et al., 2014). In addition, mefloquine was active against *Opisthorchis viverrini in vitro* and in hamsters (Keiser et al., 2009b), but not in human patients (Soukhathammavong et al., 2011). Against nematodes, mefloquine was active *in vitro* against adults and microfilariae of *Brugia patei* and *B. malayi* (Walter et al., 1987), against *Onchocerca gutturosa* (Townson et al., 1990), against microfilariae of *Loa loa* (Njouendou et al., 2018), and against *Mansonella perstans* microfilariae (Njouendou et al., 2019). Overall, mefloquine shows activity against a variety of helminth parasite species.

### 4.1 Activity of mefloquine against *E. multilocularis* metacestodes

Initially, mefloquine was tested against *in vitro* grown *E. multilocularis* metacestodes. After only 2–6 h of incubation of metacestodes in 24 μM mefloquine, a strong detachment of the GL from the LL was apparent as observed by light microscopy and scanning electron microscopy (Küster et al., 2011). Transmission electron microscopy confirmed these findings and demonstrated a time-dependent depletion of glycogen storage cells in the GL, and loss of microtriches as well as of the overall structural integrity of the parasite tissue. The PGI-assay revealed that the effects on metacestodes were dose-dependent, with an estimated EC_50_ for mefloquine of >30 μM, and no difference was observed between the (+)- and the (−) -erythro-enantiomers of mefloquine ((Küster et al., 2011; Stadelmann et al., 2011), Table 1). The IC_50_ against extracted *E. multilocularis* GL cells was calculated to be 13.8 μM in the CellTiter Glo assay ((Stadelmann et al., 2016), Table 1). Further, mefloquine-treated metacestodes from *in vitro cultures* were injected into Balb/c mice, to assess the viability of the parasite. In all 5 mice that had received *E. multilocularis* material pre-treated at 24 μM mefloquine for 10 days *in vitro*, no parasite growth was observed after 5 months of incubation. In contrast, when the parasites were pre-treated only at 12 μM mefloquine, the parasite recovered (Küster et al., 2011). The minimal concentration to exert parasiticidal effects *in vitro* was 50 μM according to the Alamar Blue vesicle viability test ((Stadelmann et al., 2016), Table 1). It was thereby proven that mefloquine has the potential to act parasiticidally against *E. multilocularis* metacestodes, although only at comparably high concentration.

**Table 1:** Shows the efficacy of mefloquine, MMV665807, and niclosamide against *E. multilocularis* metacestodes and GL cells. Given are the half-maximal concentrations of metacestode damage (PGI-assay) and GL cell viability (CellTiter Glo assay), as well as the minimal inhibitory concentrations (MIC) for the metacestode viability assay (Alamar blue assay). Values represent means from three independent experiments and respective standard deviations. Data concerning mefloquine and MMV665807 were previously published (Stadelmann et al., 2011, 2016). Data concerning niclosamide is unpublished (R. Rufener, Institute of Parasitology, Bern, Switzerland).

	Mefloquine		MMV665807		Niclosamide	
	Mean (μM)	SD	Mean (μM)	SD	Mean (μM)	SD
Metacestode damage (EC_50_)	>30	–	1.2	1.6	0.085	0.031
Metacestode viability (MIC)	50	–	1.6	–	0.3	–
GL cell viability (IC_50_)	13.8	0.33	0.6	0.37	0.111	0.008

Subsequently, mefloquine was tested in murine AE models for its efficacy. In the secondary infection model, mefloquine, applied at 25 mg/kg twice per week during 8 weeks by intraperitoneal injection, was as active as the standard ABZ treatment (200 mg/kg/day for 8 weeks (Küster et al., 2011)), but was not active when applied orally. However, when applied orally at 100 mg/kg twice per week for a duration of 12 weeks, mefloquine efficacy was similar to ABZ treatment of treatment (Küster et al., 2015). The reduction in parasite burden was hereby similar to 5 dosages of 200 mg/kg ABZ per week. At lower dosages mefloquine was not active (Küster et al., 2015). When re-injecting this parasite tissue into new mice, however, the parasite re-grew, also from the highest dosed treatment group, implying that at a treatment dose of 100 mg/kg twice per week, mefloquine was not fully parasiticidal against *E. multilocularis* metacestodes (Küster et al., 2015). In the primary (egg) infection model, treatment of mice with 100 mg/kg mefloquine twice per week during 12 weeks reduced liver lesion numbers, as assessed by visual inspection and confirmed by PCR (Rufener et al., 2018b). The reduction was slightly lower when mice were treated with 200 mg/kg ABZ for 5 days per week (Rufener et al., 2018b). However, this result should be treated with caution, since in that experiment the infection rate was relatively low. Taken together, treatment with 100 mg/kg mefloquine twice per week led to a reduced parasite mass/liver lesion number in mice, both in the primary as well as the secondary infection model. Therefore, the mefloquine plasma levels were assessed by HPLC and modelled in a standard two compartment pharmacokinetic model with first-order absorption from mice treated with mefloquine at 100 mg/kg twice per week against primary AE. An increase of mefloquine-levels over time was observed in the plasma of all mice, with C_min_ of 1.2 μg/mL and C_max_ of 2.6 μg/mL being reached to 90% after a treatment over 12 weeks (Rufener et al., 2018b). These levels are close to concentrations achieved in humans during long-term weekly dosage of 250 mg in malaria prophylaxis. Thus, this already licenced drug could possibly be active in treatment against human AE. However, data on cyst penetration and mefloquine concentrations reached in cysts, a major obstacle in the current AE treatment, is lacking to date.

### 4.2 The mode of action of mefloquine against *E. multilocularis*

The mode of action of mefloquine in *Plasmodium* involves the inhibition of hemozoin formation, a crucial step in heme degradation, and thereby leading to the accumulation of toxic heme (Egan et al., 1994). Others have demonstrated the *Plasmodium* 80S ribosomal subunit, and thus protein synthesis, to be targeted by mefloquine (Wong et al., 2017). In adult schistosomes, a similar mode of action was described (Corrêa Soares et al., 2009). In addition, the glycolytic enzyme enolase was identified as a functional target in schistosomes (Manneck et al., 2010). Further described targets are the PI3K/Akt/mTOR signalling pathways in gastric and cervical cancer cells (Liu et al., 2016; Li et al., 2017), inhibition of autophagy and induction of apoptosis in breast and colorectal cancer cells (Sharma et al., 2012; Xu et al., 2018), gap junction channels in neuroblastoma cells (Cruikshank et al., 2004), cholinesterases of murine neurons (Lim and Go, 1985; McArdle et al., 2005; Zhou et al., 2006), and non-receptor tyrosine-kinase 2 in rat brains (Milatovic et al., 2011). Especially the latter of the here mentioned targets could play a role in the described neuropsychiatric side-effects mefloquine may induce in some patients. To identify potential mefloquine targets in *E. multilocularis* metacestodes, an affinity chromatography was performed, and *E. multilocularis* ferritin was identified as a mefloquine-binding protein (Küster et al., 2015). It is notable that enolase in schistosomes, haemoglobin in *Plasmodium* and schistosomes, and ferritin in *Echinococcus*, are all metalloproteins and preferentially affected by mefloquine. *In vitro* screening of a limited series of mefloquine analogues against *E. multilocularis* metacestodes was carried out to possibly identify derivatives with lower cytotoxicity and/or higher anti-parasitic activity. However, thus far none of these analogues exhibited improved activity compared to mefloquine (Rufener et al., 2018b). Derivatives will now be used to specifically identify mefloquine-interacting target proteins from *E. multilocularis*. Nevertheless, the testing of analogues allowed for a limited SAR study, which revealed that activity of mefloquine is highly dependent on the presence of an amino group-containing residue at position 4 and the trifluoromethyl residue on position 8 of the quinoline structure (Rufener et al., 2018b). This is in line with the anti-malarial activity of mefloquine and it implies that the mode of action in *E. multilocularis* might be similar to the one against *Plasmodium*.

## 5 Repurposing of anti-malarial drugs against AE

Apart from mefloquine, several anti-malarials have been shown to be efficacious against parasitic helminths (Panic et al., 2014), and some of them were tested for their efficacy against *E. multilocularis* metacestodes: artesunate and semi-synthetic derivatives were active *in vitro*, but not in the murine AE model (Spicher et al., 2008b), synthetic amino-ozonides were partially active against metacestodes *in vitro* (Küster et al., 2014a), and atovaquone was efficacious *in vitro* and in experimentally infected mice (Enkai et al., 2020). Stadelmann et al. carried out *in vitro* screening of the Medicines for Malaria Venture (MMV) malaria box, an open-source collection of 400 compounds with proven *in vitro* activity against *P. falciparum* (Stadelmann et al., 2016). A highly interesting drug candidate, MMV665807, was identified that exhibited an EC_50_ against metacestodes of 1.2 μM (PGI assay). The IC_50_ for cultured *Echinococcus* GL cells was 0.6 μM (CellTiter Glo assay), and the minimal concentration required for parasiticidal activity was 1.6 μM ((Stadelmann et al., 2016), and own, unpublished results, Table 1). Thus, MMV665807 was more than 10 times more efficacious *in vitro* than mefloquine. Unfortunately, when assessed in the secondary infection model in mice, neither oral nor intraperitoneal application of MMV665807 resulted in any reduction of metacestode burden (Stadelmann et al., 2016). Possibly, novel formulations, which increase the plasma levels of MMV665807 could lead to better *in vivo* efficacy. MMV665807 is a salicylanilide-derivative, which is similar to the commercially available niclosamide, an anthelmintic active against adult stages of various tapeworms as well as experimentally promising against Parkinsons disease, diabetes, viral and microbial infections, and different types of cancer (Kadri et al., 2018; Haby et al., 2020). Therefore, also the *in vitro* activity of niclosamide was compared to MMV665807, and niclosamide showed an even better profile against *E. multilocularis* metacestodes *in vitro* (Table 1). Niclosamide, however, is poorly absorbed *in vivo*. Therefore, current studies involve the testing of novel formulations of niclosamide against murine AE.

## 6 Conclusion

Novel - and most importantly - improved options for the chemotherapeutical treatment of AE as an alternative to the currently applied BMZs are actively investigated. A major factor that has accelerated the search for such compounds is the availability of efficient and reliable culture methods for metacestodes as well as for isolated GL cells. This has enabled researchers to carry out medium-throughput screenings using assays such as the PGI, Alamar blue, and CellTiter Glo, all assays that are relatively inexpensive, reliable, and easy to handle. For neglected diseases in general, drug repurposing has identified anti-malarials as important resources for potential drug candidates. Among those, compounds that affect the mitochondrial complex III of the electron transport chain have emerged as interesting drug candidates, including atovaquone, and ELQ-400. In addition, *in vitro* and *in vivo* studies have shown that mefloquine could be an interesting alternative to be used in cases where BMZ toxicity is a major obstacle or patients are not responding to BMZ therapy. While the use of mefloquine could present neurological problems, derivatisation of this compound might provide a solution. Further studies on the exact mode of action of mefloquine and on cyst penetration could lead to insights that provide patients with an additional treatment option.

The significant advances in our knowledge on *Echinococcus* biology at the molecular level, especially the more recent studies on the exchange of metabolites at the host-parasite interface, has identified novel drug targets, among them the TDH and MD pathways, which are both absent in humans but are important as energy-generating mechanism in these helminths. In the past, both pathways in *Echinococcus* have mainly been studied from a biochemical perspective, but there are not many studies that investigated the potential of MD with respect to chemotherapy. Further studies will aim at targeting these pathways using drugs that act specifically against components of this pathway. For many compounds, a major problem has been that promising *in vitro* efficacy cannot be translated into activity *in vivo*, a problem that could be potentially overcome by generating chemically modified derivatives, different drug formulations, or the use of pro-drugs with improved pharmacokinetic profiles. Importantly, the recent findings on the efficacy of atovaquone in the mouse model are promising. Whether this can be translated to the situation in humans remains to be verified. However, the major concern is funding. Even though compounds are readily being made available from interested parties, drug-screening efforts need to be financed. It is important to increase public awareness about AE and its implications for our society. Only then it will be possible to generate sufficient resources to actually carry out the research that is necessary to properly evaluate chemotherapeutically promising compound classes in the future.

## Funding

This work was supported by the 10.13039/501100001711Swiss National Science Foundation (310030_192072, and 31003A_179439), and the 10.13039/501100000921COST CM1307.

## Declaration of competing interest

None.
